# Drug Repurposing: The Mechanisms and Signaling Pathways of Anti-Cancer Effects of Anesthetics

**DOI:** 10.3390/biomedicines10071589

**Published:** 2022-07-04

**Authors:** King-Chuen Wu, Kai-Sheng Liao, Li-Ren Yeh, Yang-Kao Wang

**Affiliations:** 1 Department of Anesthesiology, Chang Gung Memorial Hospital, Chiayi 61363, Taiwan; kingwutw@cgmh.org.tw; 2 Department of Nursing, Chang Gung University of Science and Technology, Chiayi 61363, Taiwan; 3 Department of Pathology, Ditmanson Medical Foundation, Chia-Yi Christian Hospital, Chiayi 60002, Taiwan; carl.liao@gmail.com; 4 Department of Anesthesiology, E-Da Cancer Hospital, Kaohsiung 82445, Taiwan; 5 Department of Medical Imaging and Radiology, Shu-Zen College of Medicine and Management, Kaohsiung 82144, Taiwan; 6 Department of Cell Biology and Anatomy, College of Medicine, National Cheng Kung University, Tainan 70101, Taiwan

**Keywords:** anesthetics, anti-cancer, re-purposing, anti-proliferation, apoptosis, signaling

## Abstract

Cancer is one of the leading causes of death worldwide. There are only limited treatment strategies that can be applied to treat cancer, including surgical resection, chemotherapy, and radiotherapy, but these have only limited effectiveness. Developing a new drug for cancer therapy is protracted, costly, and inefficient. Recently, drug repurposing has become a rising research field to provide new meaning for an old drug. By searching a drug repurposing database ReDO_DB, a brief list of anesthetic/sedative drugs, such as haloperidol, ketamine, lidocaine, midazolam, propofol, and valproic acid, are shown to possess anti-cancer properties. Therefore, in the current review, we will provide a general overview of the anti-cancer mechanisms of these anesthetic/sedative drugs and explore the potential underlying signaling pathways and clinical application of these drugs applied individually or in combination with other anti-cancer agents.

## 1. Introduction

Cancer refers to a chronic disease arising from the transformation of normal cells into tumor cells that are capable of dividing infinitely and are capable of migrating and infiltrating to destroy our normal tissues. Recent study has indicated that in 2020, there were an estimated 19 million new cancer cases and around 10 million cancer deaths worldwide [1]. Among 36 cancers, breast, lung, colorectal, prostate, and skin cancers are the five most common types of cancer, whereas lung, colorectal, liver, stomach, and breast cancers are the most common causes of cancer death [1]. In the United States, cancer is ranked as the 2nd leading cause of death disease in 2020 [2]. The diversity of cancer formation in different tissues results in the problem of the treatment since the molecular control of cancer progression in different tissues may be varied. This makes cancer treatment more challenging due to the diverse molecular mechanisms underlying the control of proliferation, cell cycle regulation, anti-apoptosis, and metastasis of different types of cancer. In the past, there were only limited treatment strategy options for cancer patients to choose for decades, including surgical resection, chemotherapy, and radiotherapy, which these strategies can apply alone or in combination [3,4]. There are also shortcomings of these conventional treatment strategies. For example, the surgical resection can only be used at an early stage of several solid cancers, whereas the inability of chemotherapeutic drugs and radiotherapy to distinguish between normal and cancer cells and eventually results in significant off-target effects and drug toxicity to the normal tissues. While these conventional treatment strategies being used for a long time, the emergence of novel therapeutic strategies in cancer research has led to a new vision through the combination of several conventional treatments and novel, innovative approaches. Although these novel treatment protocols/strategies have been developed for various cancer types, the overall average 5-year survival rate for common adult cancer subtypes is around 14–56% in North America, which is still low [5]. This result means that cancer remains the leading cause of death in most countries.

It is appreciated that the process of the development of novel anti-cancer drugs is tedious and requires lots of investment, in vitro and in vivo experiments, and subsequent clinical trials before being approved by the Food and Drug Administration (FDA, Silver Spring, MD, USA). Traditionally, it is estimated that the newly developed drug takes at least 10–15 years and requires a budget of billion dollars for research and development before entering the pharmaceutic market. Unfortunately, the success rate of getting an effective drug into clinical use is very low. This verbose research period and high investment cost make the anti-cancer drugs quite expensive and unaffordable for most cancer patients [6]. For example, in 2011, the US FDA approved several new exciting classes of drugs, including the immune checkpoint inhibitor ipilimumab (Yervoy) for malignant melanoma, and the cost of these new therapies broke the $100,000 barrier. By 2014, the average cost of a new oral medication for cancer treatment exceeded $135,000 [7]. Another example is that a newly developed drug, namely tisagenlecleucel (Kymriah), a chimeric antigen receptor (CAR) T-cell therapy for the treatment of lymphoblastic leukemia, is the most expensive drug for cancer therapy ($475,000/per person). This makes cancer treatment become a tremendous financial burden for most cancer patients. Given the long development times, costly, and low clinical success rate of conventional anti-cancer drug development, this can also dramatically increase the financial burden of the pharmaceutical companies. Other economically affordable new drug development strategies are needed. Recently, drug repurposing has become a rising research field to give a known drug a new purpose, such as anti-cancer activity, by coupling the preexisting mechanisms of these known molecules with novel anti-cancer activity. In combating cancer, it is believed that this drug repurposing represents a promising strategy of novel therapeutic strategy which can provide safe, affordable, sustainable, and effective treatment options for cancer patients.

Drug repurposing, also known as drug repositioning, is a strategy to identify new medical treatments from existing licensed drugs rather than developing new molecules. The rationale of drug repurposing is to take advantage of the existing data on drug safety, toxicity, and the ready availability of these licensed drugs to reduce the time frame and drug cost during drug development [8]. These repurposing drugs include molecules that are already in use but also molecules that are shelved, abandoned, or withdrawn due to their unexpected off-target performance, as well as their primary designated performance is not as expected [8]. A previous study elicited by Pantziarka et al. established a drug repurposing database, namely ReDO_DB, which is a curated list of original non-cancer drugs with demonstrated evidence of anti-cancer activity by using the data from peer-reviewed research reports, medical case reports, observational studies, and clinical trials [9,10]. They found that as of August 2021, there were 356 candidate drugs from ReDO_DB that showed potential anti-cancer activity. Among these 356 repurposing drugs, 164 have been involved in clinical trials of anti-cancer studies [10]. These impressive research projects on drug repurposing provide hope for the treatment of cancer and also provide clues for some of the unmet needs for cancer therapy.

As described above, the most common and primary treatment strategy to remove cancer lesions is surgical resection at an early stage of disease progression. During the surgery, anesthesia is required for the surgical treatment, which produces analgesia and amnesia, and reduces patients’ anxiety and pain. Though the purpose of surgical procedures is to cure the disease, it also presents a risk factor for cancer metastasis since the cancer cells can be spread to distal organs during the surgical procedure through circulation/lymphatic systems, resulting in distal organ metastasis [11]. Whether these pre-operative anesthetics can help reduce cancer progression, recurrent, and metastasis has become a potential treatment strategy in cancer therapy. In addition, several reports have also indicated the potential involvement of anesthetics and anesthetic procedures in cancer treatment. According to ReDO_DB, a brief list of anesthetic/sedative drugs, such as haloperidol, ketamine, lidocaine, midazolam, propofol, and valproic acid, are shown to possess anti-cancer activity [9]. Thus, in this review, we will summarize the latest evidence regarding the above-listed potential anesthetic/sedative drug repurposing on cancer biology, their underlying molecular mechanisms, and clinical outcomes.

## 2. Repurposing Anesthetic/Sedative Drugs into Anticancer Treatment

### 2.1. The Anticancer Ability of Benzodiazepine Derivative Midazolam

**Figure 1:**
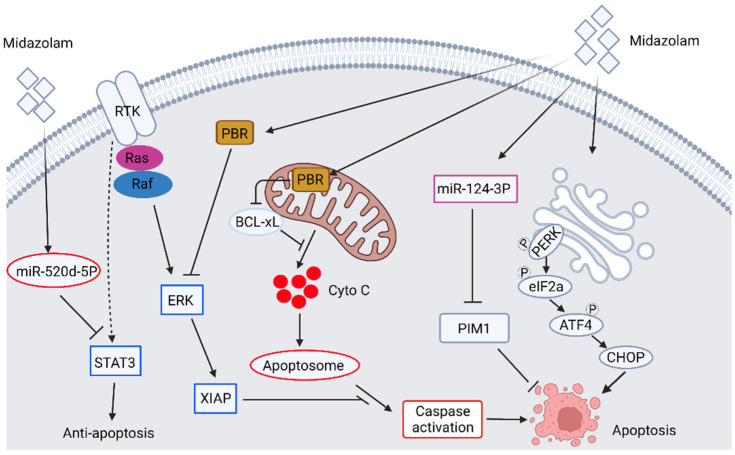
Schematic illustration of midazolam-induced apoptotic signaling in cancer cells. The figure is made by an online tool at BioRender.com (accessed on 14 June 2022).

Benzodiazepines (BDZs) are a class of drugs primarily used for general anesthesia, treating anxiety, amnesia, hypnosis, centrally-mediated muscle relaxation, and anticonvulsant activity [12]. The primary action of these BDZs is due to the potentiation of the gamma-aminobutyric acid (GABA) receptor and results in neural inhibition in the central nervous system [13]. There are three types of GABA receptors, namely GABA_A_, GABA_B_, and GABA_C_. Among them, the GABA_A_ receptor is mainly responsible for neural inhibition in the central nervous system [14]. BDZ binds to the BDZ site on the GABA_A_ receptor and results in potentiation of agonist-mediated activation of the GABA_A_ receptor. Therefore, the concentration of GABA required to activate the GABA_A_ receptor is decreased [15]. This BDZ-mediated GABA_A_ receptor potentiation increases the conductance of chloride ions and results in the hyperpolarization of postsynaptic neurons to generate an inhibitory response to these neurons. Subsequently, these neurons will not respond to further neurotransmitter stimulation. In addition to central nervous effects, BDZ can also bind to peripheral benzodiazepine receptor (PBR)/mitochondria translocator protein (TSPO) on peripheral tissues. PBR/TSPO is an 18 KDa protein located on the outer mitochondria membrane of cells and ubiquitously expressed throughout the body [16,17]. On the other hand, studies have also demonstrated the subcellular localization of PBR in the perinuclear area and nucleus [18], in the plasma membrane, and in the membrane of organelle of various cell types [19,20]. PBR is distinct in structure, ligand binding, pharmacological response, and tissue distribution compared with the central BDZ binding pocket on the GABA_A_ receptor [21]. The various subcellular localization of PBR may associate with the protein function in which PBR is involved in numerous physiologic and pathophysiologic functions. For example, PBR is involved in cell proliferation [18,22,23], steroidogenesis [24,25], regulation of mitochondrial membrane potential [21,26,27], regulation of endocrine function [24,28], and apoptosis [26,29,30]. Given the involvement of PBR in the induction of apoptosis, the application of PBR ligand for the induction of cancer cell apoptosis may be a potential target for treating cancer. Indeed, treatment of midazolam (Dormicum^®^), a short-acting BDZ derivative, with anxiolytic, sedative, hypnotic, muscle relaxant, and anticonvulsant effects, is shown to affect cancer cell behavior. Midazolam induces the apoptosis of mouse Leydig tumor cell line [31,32], non-small cell lung carcinoma cell lines (NSCLC) [33], leukemia and colon cancer cells [34], hepatocellular carcinoma cells (HCCs) [34], and lymphoma and neuroblastoma cells [35] as well as inhibiting cancer cell proliferation [31,32,33,34,35,36]. Exposure of these cancer cells to midazolam induces the activation of intrinsic caspase cascade [31,32,33,34,36], down-regulation of phosphorylated ERK [31], anti-apoptosis protein B-cell lymphoma extra-large (BCL-xL), and X-linked inhibitor of apoptosis protein (XIAP) [33]. The midazolam-induced cancer cell apoptosis can be observed not only in the in vitro model but also in the in vivo animal model [34]. Other than these traditional apoptotic pathways, midazolam can also induce apoptosis through novel signaling. So et al. have discovered the upregulation of several endoplasmic reticulum stress markers, such as p-EIF2a, ATF3/4, CHOP, and the ratio of LC II/I upon treatment of midazolam in mouse Leydig tumor cell line [32]. Jiao et al. have discovered that midazolam induces apoptosis of A549 lung cancer cells through the targeting of signal transducer and activator of transcription 3 (STAT3) by microRNA miR-520d-5p [33]. This midazolam-induced expression of microRNA miR-520d-5p binds to the 3′ untranslated regions of STAT3, leading to the inhibited expression of STAT3. The down-regulated STAT3 results in the upregulation of Bax, caspase activation, downregulation of Bcl-2, and then induction of apoptosis. Qi et al. have also demonstrated that upon treatment of midazolam, the cell proliferation, colony-forming ability, cell migration, and invasion of hepatocellular carcinoma (HCC) are inhibited, whereas the levels of microRNA miR-124-3p are upregulated. They also determine that overexpression of miR-124-3p mimics decreases tumor size, whereas miR-124-3p inhibitor reverses midazolam’s inhibitory effect in vivo. They further discover that midazolam-induced upregulation of miR-124-3p targets an anti-apoptotic protein PIM-1 and results in the degradation of PIM-1, which in turn leads to the induction of apoptosis [35]. The midazolam-induced apoptotic signaling pathways in cancer cells are summarized in Figure 1. Other BDZ derivatives, such as olanzapine, induce sensitization of lung and pancreatic cancer stem cell lines to the treatment of chemotherapeutic agents by attenuating the expression of survivin, an anti-apoptotic protein [37]. Another benzodiazepine derivative, the remimazolam, induces apoptosis of human glioblastoma multiforme (GBM) cell lines by increasing caspase activation, decreasing protein levels of Bcl-2, XIAP, and NF-κB [38]. The above results demonstrate that the benzodiazepine derivatives induce cell apoptosis and inhibit cell proliferation through different signal pathways in different cancer cells.

Besides inhibiting cancer cell proliferation and induction of apoptosis, midazolam is also found to inhibit cancer cell invasion and metastasis. We and other labs have discovered that treatment of midazolam inhibits cancer cell proliferation, migration [39,40], invasion [39,40], and epithelial-mesenchymal transition [40]. These inhibitory effects of midazolam on cancer cell proliferation and metastasis are mediated by PBR, as the treatment of PBR antagonist reverses these inhibitory effects of midazolam [40]. Seo et al. discover that midazolam inhibits hyperglycemia-induced melanoma lung metastasis. Midazolam treatment does not affect the viability and migration of melanoma cells. Still, it decreases vascular endothelial growth factor (VEGF)/high glucose-induced endothelial cell migration, ROS generation, and vascular permeability, decreasing lung metastasis of melanoma cells. Interestingly, this inhibitory effect of midazolam on vascular endothelial cells is mediated by GABA signaling but not PBR, as the performing antagonist of a GABA signal reverses the inhibitory effects of midazolam but not the PBR antagonist [41]. In addition, midazolam can exert its tumor-suppressing effects by increasing cisplatin sensitivity in cisplatin-resistant non-small cell lung cancer cell lines (CR-NSCLC). This midazolam-enhanced cisplatin sensitivity is mediated by upregulation of miR-194-5p, where the application of a miR-194-5p inhibitor abrogates the inhibitory effect of midazolam on cell proliferation and viability in cisplatin-treated CR-NSCLC cells [42]. The downstream target of miR-194-5p is hook microtubule-tethering protein 3 (HOOK3), known as a microtubule-associated adaptor protein, and the function of HOOK3 involves intracellular membrane trafficking [43,44]. Overexpression of HOOK3 in CR-NSCLC reverses midazolam-enhanced cisplatin sensitivity, suggesting the midazolam-induced miR-194-5p/HOOK3 axis is involved in the progression of cancer drug resistance. This observation can not only be observed in the in vitro experiment but also in the xerograph model. In addition, using a mouse model of pancreatic ductal carcinoma (LSL-Kras^G12D/+^; Trp53^flox/flox^; Pdx-1^cre/t^ transgenic mice), Oshima et al. have demonstrated the therapeutic effects of midazolam in this animal model. The results show that midazolam treatment decreases tumor size, expression of Ki-67, cyclins, and cyclin-dependent kinases. The tumor-associated neutrophils, macrophages, polymorphonuclear myeloid-derived suppressor cells, and pro-inflammatory cytokines are reduced by midazolam treatment. These inhibitory effects of midazolam can be reversed by the co-treatment of its antagonist PK11195 [45]. This study suggests that midazolam can not only be applied to the xenograft model of cancers but also applied to treat cancer that bears genetic mutations.

The above studies demonstrate that the anesthetic/sedative drug BDZ and its derivatives, such as midazolam, can be repurposed to inhibit cancer progression. Midazolam is shown to inhibit cancer cell proliferation, migration, metastasis, and induction of apoptosis through various pathways, or in combination with other traditional cancer chemotherapeutic agents for treating cancer. The signaling pathways involved in midazolam-inhibited cancer progression are quite different in various cancers. Since most of the studies focus on the inhibition of cancer proliferation and induction of cancer cell apoptosis, the impact of midazolam on other cancer behavior, such as cancer invasion, EMT, and metastasis, needs further investigation. In addition, though there have been numerous reports showing midazolam in treating cancer cells, only a limited number of papers have demonstrated the antitumor effects of midazolam in animal models [34,39,41]. Further investigation using different animal models should be conducted to address the effect of midazolam on anti-cancer potential in vivo. Furthermore, there are 43 clinical trials of phase I/II in recruiting or completion stages using midazolam in combination with other chemotherapeutic molecules in different cancer therapies (clinicaltrials.gov (accessed on 24 April 2022). Searching keywords: midazolam, cancer). However, most of these clinical trials focused on drug-drug interaction to investigate the potential anti-cancer drugs interacting with the substrate (midazolam) of cytochrome P450 3A. Others focus on the sedative/pain relief functions of midazolam. None of these trials investigate the anti-cancer effect of midazolam in cancer treatment. Further investigation of the roles of treatment of midazolam alone or combined with other chemotherapeutic molecules for anti-cancer purposes is crucial in translating the observation from laboratory to clinical use.

### 2.2. The Potential Antipsychotic Agents in Anticancer Studies: Role of Haloperidol and Its Derivatives

Haloperidol belongs to a class of butyrophenones of antipsychotic agents and is a potent central nervous system dopamine receptor antagonist by blocking the postsynaptic dopamine receptor in neurons [46]. Haloperidol does not have antihistamine or anticholinergic properties and is quite effective against hallucinations due to its direct inhibitory effect on central dopaminergic signaling. Therefore, the primary purpose of the application of haloperidol is for the treatment of symptoms associated with schizophrenia [47]. Given that these antipsychotic agents are permeable to the blood-brain-barrier, it is likely that these drugs can be potentially repurposed for other CNS disease treatments, such as treatment of neuroblastoma/glioblastoma multiforme (GBM). Gassó et al. have discovered that treatment with haloperidol, risperidone, and paliperidone alone or in combination shows that only haloperidol can induce activation of caspase 3 and apoptosis of the human neuroblastoma cell line SN-K-SH. On the other hand, treatment of risperidone and paliperidone reduced this effect [48]. Though risperidone does not show a significant cytotoxicity effect in the in vitro study, a case report has revealed that in a schizophrenic patient with GBM, treatment of risperidone resulted in an elongated survival of 6.5 years [49] compared with a median survival time of 15 months and 5-year survival of ~5% after initial diagnosis of GBM [49]. This result, unlike the in vitro study, suggests the potential anti-cancer effect of risperidone in treating GBM. The mechanisms of anti-cancer effects of these antipsychotic agents, such as haloperidol, induce cell cycle arrest at the G2/M phase, induction of apoptosis and activation of caspase, and inhibition of cell migration [50]. A combination of haloperidol treatment with temozolomide (TMZ) and radiotherapy can further decrease cell survival in GBM [51]. In addition, another study also demonstrates that haloperidol treatment disrupts Akt signaling, resulting in the dephosphorylation of Bcl-xs. This dephosphorylated Bcl-xs then interacts with the mitochondrial voltage-dependent anion channel and causes the release of cytochrome C to activate caspase cascade and apoptosis [52]. With such anti-proliferation and anti-metastatic ability of these antipsychotic molecules, several studies have performed these molecules or their derivatives to treat various cancers. Certain analogs of haloperidol, such as 4-(4-(4-chlorophenyl)-1,4-diazepan-1-yl)-1-(4-fluoro -phenyl) butan-1-one (SYA013), are shown to inhibit the proliferation of triple-negative breast cancer MDA-MB-231 cells and induces apoptosis of MDA-MB-231 cells through the intrinsic apoptotic pathway [53]. In addition, in vinblastine-resistant (VR) human leukemic cell lines, haloperidol treatment at a non-toxic dose is found to enhance the cytotoxic effect of VR cells but not in the parental cell line. This effect of haloperidol in reversing VR is mediated by competitively inhibiting drug binding to P-glycoprotein to reverse multiple drug resistance and then potentiates anti-cancer ability [54].

Although several studies have demonstrated the potential anti-cancer ability of these antipsychotic agents, the evidence and detailed molecular mechanisms are still limited. On the clinical trials website, we found six trials of phase I/II in recruiting or completion stages using haloperidol alone or in combination with other drugs to control the symptoms of delirium and relief of nausea and vomiting in patients with advanced cancer. None of them associate anti-cancer therapy. Further studies to investigate the anti-cancer effects and underlying molecular mechanisms using haloperidol and its similar drugs alone or in combination with both in vitro models and in vivo models are needed.

### 2.3. The Anti-Cancer Ability of Ketamine

Ketamine is an antagonist of the N-methyl-D-aspartate (NMDA) receptor that is primarily used in the induction and maintenance of anesthesia. It is now widely used as an anesthetic, sedative, and analgesic for various clinical purposes [55]. It induces a trance-like state of dissociative anesthesia, which can also provide pain relief and amnesia. Therefore, ketamine is prescribed to those cancer patients for pain management. Nevertheless, one of the major side effects of ketamine is neurotoxicity, as ketamine induces neuronal apoptosis, impairs the learning and memory of adolescent rats [56], and induces hippocampal neurodegeneration and long-term cognitive impairment in developing rats [57]. In addition, early studies have also found that treatment of ketamine is found to inhibit cell proliferation and causes cell cycle arrest in several cells [58,59], suggesting a possible application of ketamine in the inhibition of cancer progression. Zhou et al. have found that ketamine treatment induces apoptosis of lung adenocarcinoma cells by the up-regulation of CD69 [60], a leukocyte and natural killer (NK) cell activation marker, and is shown to induce apoptosis in THP-1 cells [61]. Applying ketamine in different ovarian cancer cell lines results in cell cycle arrest, inhibition of colony-forming capacity, and induction of apoptosis. This ketamine-induced anti-cancer effect in ovarian cancer is mediated by the down-regulation of long noncoding RNA PVT1. Mechanistically, treatment of ketamine inhibits recruitment of P300/CREB binding protein (CBP) to the PVT1 promoter region. In addition, treatment of ketamine decreases the interaction between PVT1 and histone methyltransferase enhancer of zeste homolog 2 (EZH2). This interaction causes the upregulation of P57, a cell cycle inhibitor which in turn suppresses the cell cycle progression of cancer cells [62]. Furthermore, treatment of ketamine can suppress proliferation and induces ferroptosis, a type of cell death driven by iron-dependent, multiple cellular metabolic pathways [63], and apoptosis by targeting glutathione peroxidase 4 in breast cancer cells [64] and HCCs [65]. For the induction of immunosuppression in cancer treatment, some small-scale clinical trials have reported that the pre-operative intravenous infusion of ketamine only possesses minor [66] or even slight inhibitory effects [67] on cytotoxicity of NK cells. These results suggest that ketamine may not be able to enhance NK cell cytotoxicity activity to exert its anti-cancer activity. Interestingly, the detailed molecular mechanisms of anti-cancer activity of ketamine are still elusive; there is an ongoing clinical trial using ketamine to treat colorectal cancer that is currently recruiting patients (Table 1, clinicaltrials.gov (accessed on: 24 April 2022), searching keywords: ketamine, cancer). The anti-cancer effects of ketamine still require more sophisticated experimental designs and should be translated from laboratory studies to in vivo and further.

### 2.4. The Application of Lidocaine and Its Similar Drugs in the Anti-Cancer Study

**Figure 2:**
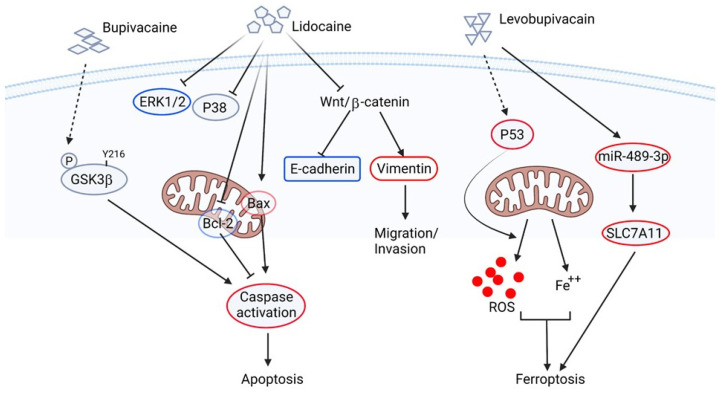
Schematic illustration of anti-cancer signaling pathways of lidocaine and its derivatives in cancer cells. The dotted line represents the pathway is proven, but the mechanism is unknown. The figure is made by an online tool at BioRender.com (accessed on 14 June 2022).

Lidocaine, also known as lignocaine, is an amide local or regional anesthetic, usually used for superficial and invasive procedures. The anesthetic action of lidocaine and its similar drugs blocks the sodium channel activity. Therefore, the neurons of the local tissues are not capable of sending a response to the CNS [68]. Besides anesthesia, this type of local anesthetic drug shows a potential off-target cytotoxic effect [69,70], which this cytotoxic effect may be an additional molecular event for anti-cancer therapy. Early study has indicated that bupivacaine, a long-acting local anesthetic, can induce apoptosis of both ovarian and prostate cancer cells. Xuan et al. have discovered that treatment of bupivacaine induces phosphorylation at tyrosine 216 residue of glycogen synthase kinase 3 beta (GSK3β). This bupivacaine-induced phosphorylation of GSK3β promotes activation of both caspase 8 and 9 in ovarian cancer cells, whereas only caspase 8 is activated and is independent of GSK3β in prostate cancer cells [71]. This in vitro study suggests that bupivacaine may possess a direct anti-cancer effect by activating apoptotic pathways against ovarian and prostate cancer. Sun et al. have also reported that the inhibitory effects of lidocaine on cell proliferation and metastasis are mediated by the Wnt/β-catenin pathway of ovarian cancer cells [72]. Similar results are also found in HCC, where lidocaine treatment causes apoptosis through the activation of pro-apoptotic protein Bax, caspase cascade, and a corresponding decrease in anti-apoptotic protein Bcl-2 through the extracellular signal-regulated kinase (ERK) 1/2 and p38 pathways. Importantly, these in vitro studies can be translated to in vivo, whereas lidocaine suppresses tumor growth and enhances tumor sensitivity to cisplatin treatment in a xenograft model [73]. Another study showed that the treatment of levobupivacaine, another aminoamide local anesthetic, inhibits the proliferation of prostate cancer cells by combined inhibition of glycolysis and oxidative phosphorylation, which in turn decreased ATP production and bioenergetic crisis, as well as reactive oxygen species (ROS) generation [74]. Since treatment of levobupivacaine regulates ROS generation, Meng et al. revealed that levobupivacaine treatment inhibits proliferation and induces apoptosis of NSCLC cancer cell lines. They also discovered that treatment of levobupivacaine upregulates ROS, iron, and Fe^2+^ in NSCLC cells which in turn induces ferroptosis [75]. This levobupivacaine-induced ferroptosis is mediated by up-regulation of P53. Mao et al. also discovered that levobupivacaine could suppress cell viability and inhibit the proliferation of gastric cancer cells by enhancing ferroptosis both in vitro and in vivo. This levobupivacaine-induced ferroptosis in gastric cancer cells is mediated by the up-regulation of microRNA miR-489-3p and targeted on a ferroptosis regulator SLC7A11 [76]. On the other hand, cancer malignancy is a multistep progression, including both genetic abnormality and epigenetic modification. Among them, the methylation-induced silencing of the tumor suppressor gene has attracted researchers’ attention in tackling cancer malignancy. Therefore, molecules associated with demethylation may be potential targets to release those silenced genes. An early report has indicated that procaine, another amide-based local anesthetic, is reported to demethylate hypermethylated CpG islands to release epigenetically silenced tumor suppressors, such as retinoic receptor beta 2. This finding explains the binding of procaine to the CpG-enriched promoter region and the growth inhibitory effect of procaine by cell cycle arrest in human cancer cells [77]. Lidocaine and similar drugs-induced anticancer activity and signaling are summarized in Figure 2.

Additionally, immunosuppression is one of the hallmarks of cancer, where the innate immunity of the host is usually suppressed during cancer progression. Therefore, activating these immune responses has become one of the therapeutic strategies for killing cancer cells. Current strategies mainly focus on the induction of T cell cytotoxic activity in which the T cell-mediated cytotoxic to cancer cells, such as immune checkpoint therapy, has become a popular focus in killing cancer cells [78]. It is unknown whether these local anesthetics can promote or synergize with these T-cell-mediated immunotherapies. However, several reports have indicated the importance of amide local anesthetics in activating Natural Killer cells (NK cells) in killing cancers. Ramirez et al. have discovered that treatment of clinically achievable plasma concentration of lidocaine can enhance the in vitro function of NK cells. These low concentrations of lidocaine treatment can induce cytotoxicity of NK cells against human leukemia cell lines, such as K562, THP-1, and OCI-AML3 [79]. Cata et al. isolated NK cells from healthy donors and patients who have undergone surgical removal of cancers and these isolated NK cells were treated with lidocaine. They found that lidocaine treatment increased levels of NK cell marker (NKGD) and cytotoxic activity against ovarian, pancreatic, and osteosarcoma cell lines in NK cells isolated from those cancer patients [80]. To test whether treatment of amide local anesthetics in cellular immunity, a small-scale clinical trial was conducted. They recruited patients who received primary breast tumor resection and then subjected them to control or 2% lidocaine infusion groups. After treatment, the percentage of NK cells in the lidocaine infusion group was significantly higher than in the control. The trends of CD3+, CD4+, CD4+/CD8+ but not CD8+ were different. These results suggest that the intravenous infusion of lidocaine may regulate the immune function of breast cancer patients during the pre-operative period [81]. However, whether this intravenous infusion of lidocaine-induced immune regulation in the treatment of cancer progression requires further investigation.

### 2.5. The Application of Propofol in Cancer Treatment

Propofol is a fast onset, short-acting intravenous injected anesthetic agent with sedative, hypnotic, and mild analgesic effects. The anesthetic mechanisms of action of propofol are similar to barbiturates and benzodiazepines, in which the function of propofol is to enhance the activity of central GABA receptors to stimulate the central inhibitory neurotransmitter response. Upon the binding of propofol to the GABA receptor, it induces chloride ion influx and results in hyperpolarization of the postsynaptic neurons, which in turn blocks further external stimuli to the neurons [82]. In addition, several reports have also indicated that propofol possesses an anti-inflammatory response [83] and perhaps stimulates the immune response in the elderly [84], which implies the possible role of propofol in the treatment of cancer through an indirect effect on cancer or the regulation of immune response. Interestingly, Ai and Wang discovered that the cytotoxic activity of natural killer cells (NK cells) in gastric patients is low. In contrast, it is enhanced in patients receiving propofol anesthesia, suggesting that propofol can enhance the cytotoxic activity of NK cells to possess better tumor-killing ability [85]. Other than regulating the inflammatory/immune response, numerous reports have focused on the anti-proliferation ability of propofol in various cancer cells. Indeed, propofol inhibits cancer cell proliferation via different signal pathways. To tackle cancer cell proliferation, the strategy of regulating oncogene/tumor suppressor genes can be focused on. In gastric cancer, propofol inhibits gastric cancer proliferation via the regulation of various microRNAs and their downstream oncogenes, such as STAT3. Bai et al. have found that propofol can increase the levels of miR-328-3p, which in turn downregulates STAT3 and its downstream genes to suppress gastric cancer cell proliferation [86]. Tabnak et al. have summarized the microRNAs regulated by propofol and their downstream target genes in various cancers [87]. In addition to proliferation inhibition, propofol treatment induces the activation of caspase and MAPK cascade but diminishes the survival signal, such as phosphorylation of Akt to induce apoptosis of mouse Leydig tumor cells [88]. Besides, propofol induces apoptosis of 5-fluorouracil (5-FU) resistant oral squamous cell carcinoma (OSCC). Treatment of propofol can reverse the resistance of OSCC to 5-FU by reducing the expression and secretion of amphiregulin, which is known to relate to the poor prognosis of cancer [89].

However, another small-scale clinical trial conducted by Lim et al. compared the effect of propofol and sevoflurane on NK cells, cytotoxic T lymphocyte (CTL) counts, and apoptosis rate in breast cancer cells after anesthesia. The results showed that both anesthetic agents did not impact the aspects of NK and CTL cells counts with apoptosis rate, including breast cancer cells in the clinical environment [90]. Together, though propofol treatment has revealed its effective anti-cancer potential in the in vitro studies, the mechanisms and downstream effectors involved in propofol-inhibited cancer progression are mostly unknown. Further studies tackling the molecular mechanisms underlying propofol-inhibited cancer progression and using propofol alone or combined with chemotherapeutic drugs in animal studies and clinical trials are needed.

### 2.6. The Effect of Valproic Acid in Anti-Cancer Therapy

**Figure 3:**
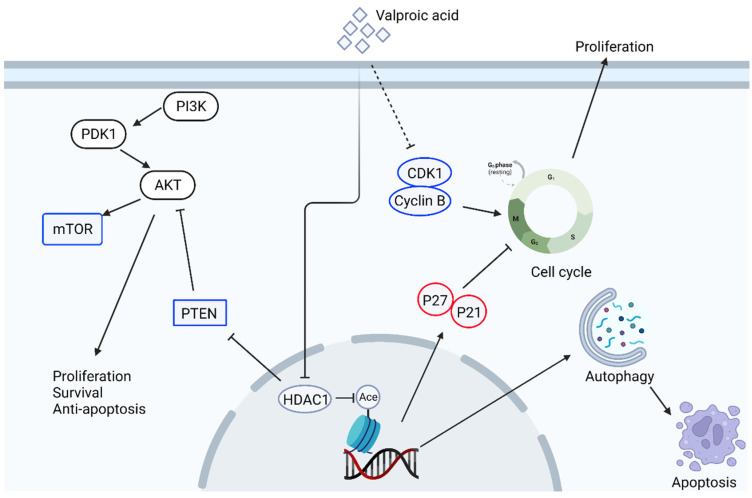
Schematic illustration of anti-cancer signaling pathways of valproic acid in cancer cells. The dotted line represents the proven pathway, but the mechanism is unknown. This figure is made by an online tool at BioRender.com (accessed on 15 June 2022).

Valproic acid (2-propylpentanoic acid, VPA) is primarily used to treat epilepsy and bipolar disease and has a broad spectrum of anticonvulsant activity [91]. The pharmacological mechanism of VPA is through the inhibition of GABA transaminase to increase the amount of GABA in the synaptic cleft and potentiate GABAergic function, which in turn inhibits the activation of postsynaptic neurons in CNS [92]. The structure of VPA is composed of a short-chain fatty acid, which can also interact with histone deacetylase (HDAC) and inhibits its function of suppressing the expression of many genes [93]. Since epigenetic modification, such as histone acetylation and DNA methylation, play an important role in cancer progression, metastasis, and recurrence, the role of these epigenetic modifiers is thought to be a potential anti-cancer target for cancer therapy. Early study has demonstrated that treatment of VPA inhibits proliferation of cervical cancer cell lines and upregulates net histone H3 acetylation and P21 expression but not P53. This VPA-induced inhibitory effect can be translated to in vivo xenograft model as tumor growth is inhibited and animal survival is improved by the treatment of VPA [94]. Similar results can be found in chronic administration of VPA in prostate cancer, as chronic administration of VPA (up to 14 days) to prostate cancer cells suppresses proliferation. This prolonged administration induces the expression of P21 and activation of the caspase cascade in both androgen-dependent and independent prostate cancer cells. This prolonged VPA treatment can also reduce cancer growth in a xenograft model [95]. Using human prostate cancer xenograft animals, Sidana et al. have also demonstrated that animals receiving VPA treatment show a decrease in tumor size, cyclin D1, and androgen receptor, but an increase in the protein levels of cell cycle inhibitor P21 and P27, and apoptotic cells [96]. In addition, the treatment of VPA inhibits proliferation and induces autophagy and apoptosis in gastric cancer cells. The VPA-induced autophagy, which leads to apoptosis, is mediated by the inactivation of HDAC1, followed by the upregulation of PTEN and inhibition of p-AKT [97]. Similar results can be observed in head and neck cancer cells, where the treatment of VPA inhibits cell growth through the upregulation of P21, subsequent upregulation of terminal differentiation, and cell senescence [98]. In Her2-positive breast cancer cells, treating VPA can also inhibit cell growth by targeting cell cycle arrest and induction of apoptosis. This VPA-induced cell cycle arrest and apoptosis are through the acetylation of heat shock protein 70 [99]. A retrospective report also summarizes the results of VPA in the treatment of ER/PR/Her2 positive or triple-negative breast cancer cells. The treatment of VPA can induce apoptosis, cell cycle arrest, inhibition of invasion and metastasis, and the modulation of immune response in breast cancer [100]. In drug-resistant cancer cells, the treatment of VPA can reverse the drug-sensitive of these cancer cells. For example, in the sorafenib-resistant HCC cell line, the Notch signaling and PI3K/Akt signaling pathway are activated in these resistant cells to avoid the sorafenib-induced anti-cancer effect. A combination of VPA with sorafenib can enhance the drug sensitivity of these cancer cells and reverse the upregulated Notch and PI3K/Akt signaling pathways [101]. In addition, in prostate cancer, drug resistance is inevitably developed in almost all patients receiving hormonal therapy. Different generations of anti-prostate cancer drugs either targeting the androgen/androgen receptor [102] or other pathways such as PI3K/Akt/target of rapamycin (mTOR) [103] are developed. In these drug-resistant cells, cell cycle proteins such as cyclin B and cell cycle-dependent kinase 1 (cdk1), together with Akt/mTOR signaling, are upregulated. Unfortunately, the resistance can still be observed in these drug-resistant prostate cancers. Treatment of VPA suppresses the growth of drug-resistant prostate cancer cells, expression of cyclin B/cdk1, phosphorylation of mTOR, and upregulates acetylation of histones H3 and H4. Mechanistically, VPA restores the sensitivity of prostate cancer cells to the chemotherapeutic drug by down-regulating cdk1, cyclin B, and Akt/mTOR signaling [104]. The anti-cancer signaling pathways of VPA are summarized in Figure 3.

Due to its inhibitory effect on HDAC, which is shown to be involved in cancer progression, metastasis, and recurrent, numerous reports have demonstrated the potential anti-cancer properties of VPA [100,105,106,107,108]. Indeed, there have been reports showing the application of VPA in clinical trials. For example, a clinical trial conducted by Iwahashi et al. in Japan investigated the co-treatment of valproic acid in combination with S-1 on advanced pancreatic cancer. The result shows that the combination therapy in treating advanced pancreatic cancer provides a high rate of tumor control and acceptable drug toxicity [109]. In addition, there are at least 40 different clinical trials in either activating, recruiting, or in completed status using VPA together with other chemotherapeutic agents to treat various cancers (clinicaltrials.gov (accessed on 25 April 2022), searching keywords: valproic acid, cancer). However, there are also reports showing the different results of VPA in cancer treatment. Zhang et al. have revealed that the treatment of VPA does not affect cell proliferation but induces EMT-like properties of breast cancer cells. This VPA-induced EMT is mediated by the induction of EMT-associated transcription factors Snail and Zeb1 [110]. Another clinical trial conducted by Chu et al. performed VPA in combination with 5-aza-2′-deoxycytidine in treating non-small cell lung cancer. Unfortunately, this combination treatment is limited by several unacceptable side effects [111]. In addition, in different types of cancer cells, such as HeLa uterine cervix carcinoma cells, 5637 bladder cancer cells, and SCC-15 squamous cell carcinoma cells, treatment of VPA induces EMT morphology and upregulation of EMT markers [112]. The above studies suggest that though VPA possesses substantial advantages in the treatment of cancer both in vitro and in vivo, the controversial treatment effects of VPA on certain types of cancer can still be observed. Further studies to access the efficacy, safety, and durability of VPA treatment alone or in combination with other chemotherapeutic agents in animal models and clinical trials of different cancer types are needed.

### 2.7. The Anti-Cancer Potential of Volatile Anesthetics

Inhalation anesthetics, including halothane, isoflurane, desflurane, sevoflurane, and others, are commonly used for the induction and maintenance of anesthesia during surgical procedures. Most of these anesthetic agents are liquid at room temperature and require vaporizers for the anesthetic procedure. These inhalation anesthetics are also commonly combined with other intravenous injected anesthetics during surgery. The action mechanisms of these inhalation anesthetics are not well known but presumably exert their function by augmenting the GABA receptors to enhance the inward flow of chloride ions and depress the neurons in CNS [113]. Several retrospective studies have indicated that inhalation anesthesia can improve the prognosis and overall survival rate of patients with breast cancer, colon cancer, ovarian cancer, and other cancers [114,115]. Indeed, treatment of sevoflurane at the concentration of 5% inhibits cell proliferation, migration, and invasion and induces apoptosis in GBM cells. This inhibitory effect of sevoflurane is through the inhibition of insulin-like growth factor-1 (IGF-1) and its downstream PI3K/AKT signaling [116]. In colon cancer, sevoflurane treatment can also inhibit cell proliferation, migration, invasion, and EMT, but stimulate autophagy and apoptosis. This sevoflurane-inhibited colon cancer progression is mediated through Raf/MEK/ERK signaling, as treating ERK activator LM22B-10 triggers Raf/MEK/ERK signaling and reverses the inhibitory effects of sevoflurane. The sevoflurane-inhibited colon cancer progression can also be translated from in vitro to in vivo [117]. Moreover, Fan et al. have found that sevoflurane treatment does not affect colorectal cancer (CRC) cell proliferation but suppresses migration and invasion. This sevoflurane-inhibited CRC migration and invasion is mediated by the upregulation of microRNA miR-203, as upregulated miR-203 inhibits phosphorylation of the ERK/MMP9 axis, leading to the inhibition of CRC migration and invasion [118]. In breast cancer cells, sevoflurane treatment inhibits cell proliferation by suppressing protein levels of cyclin D/E and up-regulation of cell cycle inhibitors, such as P21, P27, and retinoblastoma 1. The growth inhibitory effect of sevoflurane is mediated by the up-regulation of miR-203 [119]. Other studies also demonstrate that treatment of sevoflurane can alleviate the malignancy of lung cancer cells by inhibiting the hypoxia-inducible factor 1α [120]. Other inhaled agents, such as isoflurane, are shown to induce growth inhibition, apoptosis, caspase cascade, and inhibition of migration and invasion of HCCs [121].

**Figure 4:**
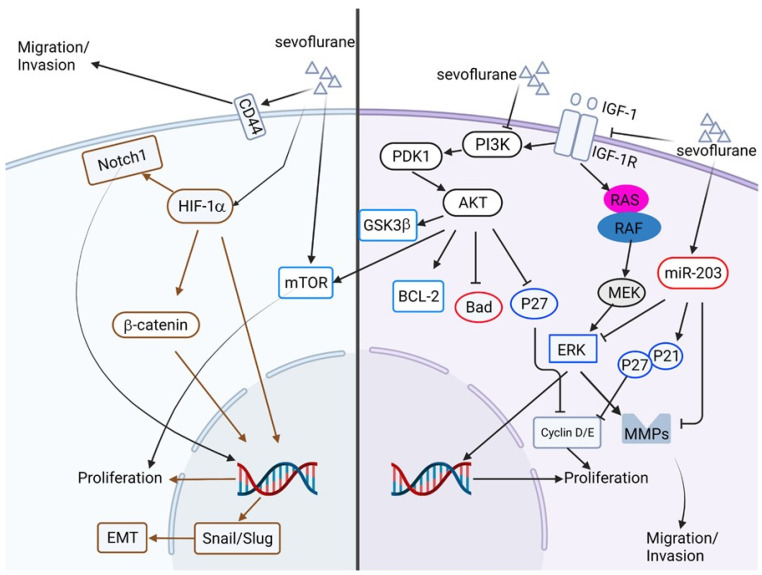
Schematic illustration of volatile anesthetics in the regulation of cancer progression. The right half represents the anti-cancer effects of sevoflurane in the treatment of various cancers. The left half represents the treatment of sevoflurane induces proliferation, migration, invasion, and EMT in certain cancers. The brown color represents the signaling pathways elicited by HIF-1α. This figure is made by an online tool at BioRender.com (accessed on 15 June 2022).

However, there is also a report showing that inhaled anesthetics, such as isoflurane and sevoflurane, inhibit immune response and play a pivotal role in cancer progression [121]. The literature shows little or no effects of these inhaled anesthetics in cancer progression [122,123]. Several reports also demonstrate contradictory results, showing the cancer-promoting effects of these inhaled anesthetics in various cancers. For example, Other than inhibition of GBM cancer progression, Shi et al. show different results that sevoflurane treatment increases glioma stem cell expansion in vitro. This sevoflurane-induced cancer stem cell expansion is mediated by hypoxia-inducible factor (HIF) [124]. Lai et al., also demonstrate sevoflurane can induce proliferation, migration, and invasion of GBM cells and is possibly mediated by the induction of cell-surface glycoprotein CD44 [125]. In addition, treatment of isoflurane promotes EMT and metastasis of bladder cancer cells through HIF-1α/β-catenin/Notch1 pathways [126]. Isoflurane treatment can promote the proliferation of squamous cervical cancer cells and is mediated through mTOR-histone deacetylase 6 signaling [127]. The signaling pathways associated with sevoflurane-regulated cancer cell progression are summarized in Figure 4.

Since NK cells are known to involve in cancer recurrence/metastasis in the pre-operative period, Tazawa et al. have demonstrated that treatment of isoflurane or sevoflurane attenuates the cytotoxicity of NK cells to cancer cells in vitro. This immunosuppression effect of isoflurane and sevoflurane to NK cell cytotoxicity is mediated, at least in part, by down-regulation of leukocyte function-associated antigen-1 [128]. Buckley et al. have drawn serum from patients who receive sevoflurane anesthesia and experienced surgery to remove breast cancer, followed by treatment with opioid analgesia and then co-culture with NK cells from healthy female donors. The results show that sevoflurane anesthesia-treated serum decreases the expression of NK cell marker CD16 and decreases the cytokines interleukin-10 (IL-10) and IL-1β [129]. However, serum from propofol anesthetized patients increases the expression of NK cell marker CD107a and the cytotoxicity ability of NK cells [130]. The above results suggest that these volatile anesthetics may possess diverse effects when applied in pre-operative anesthesia for cancer surgery or treating various cancer types. Given that these volatile anesthetics are not traditional anti-cancer drugs, it is unclear how these volatile anesthetics exert their anticancer-relevant signaling. By performing systemic analysis, such as next-generation sequencing, big data analysis can provide us with more detailed information regarding these volatile anesthetics in treating cancer progression. However, there are still reports of using sevoflurane for anti-cancer treatment, which can be found on clinicaltrials.com (Searching keywords: sevoflurane, cancer), whereas clinical trials using other volatile anesthetics for cancer therapeutic purposes are not found (Table 2).

## 3. Conclusions and Future Perspective

Despite the recent reports demonstrating the advantages of chemotherapeutic drugs in regulating relevant anti-cancer signaling in human cancer, the unacceptable side effect and potential cytotoxicity of these drugs in normal cells limited the effectiveness and safety of these chemotherapeutic drugs. Sustained, and perhaps insufficient dosage treatment of these drugs to cancer cells may cause the cancer cells to acquire drug resistance, which results in failed treatment using these standard therapeutic procedures. In order not to increase the financial burden of cancer patients, effective, patient-friendly, low toxicity, and low-cost novel anti-cancer drugs/strategies are needed. Recent studies have indicated the potential applications of these anesthetics in anti-cancer therapy. Administration of these anesthetics to cancer cells can regulate gene expression, epigenetic modification, and signal transduction, resulting in anti-cancer properties, such as anti-proliferation, induction of apoptosis, and anti-metastasis (Table 3). Other than the regulation of traditional signalings, such as cell cycle progression and caspase cascade, novel signaling pathways, such as the induction of microRNAs and long noncoding RNAs and the regulation of their downstream genes in proliferation/apoptosis controls are also found in the treatment of these anesthetics. In addition, these anesthetics show additive or synergistic effects with several traditional chemotherapeutic drugs and perhaps lower the treatment dose of these traditional chemotherapeutic drugs to reduce cytotoxicity in normal cells. However, most of these reports focus on a specific signaling pathway that leads to the inhibition of proliferation or induction of apoptosis. It is not known whether there are interactions of other signal pathways together with the specific signaling in the regulation of cancer progression upon treatment with these anesthetics. For example, several studies focus on regulating a single miRNA and its downstream targets in mediating the anti-cancer effect of anesthetics. This design may not provide a complete perspective on the anti-cancer effects of anesthetics. Therefore, a series of systemic studies such as the application of next-generation sequencing, genomic/proteomic studies, and big data analysis to obtain multidimensional views upon treating these anesthetics are needed. Indeed, such approaches can provide a global view and advantages comparing the expression pattern of genes/miRNAs/long noncoding RNAs and a better standpoint in combining the treatment of anesthetics alone or in combination with other chemotherapeutic agents in different cancer patients. Thus further conceptual refinement and search on the drug target in different cancers are crucial for bringing these experimental findings to clinical cancer treatments.

## Figures and Tables

**Table 1:** A clinical trial of ketamine in the treatment of colorectal cancer.

Status	Study Title	Condition	Interventions	Locations
Recruiting	The effect of ketamine on immune function and prognosis in patients undergoing colorectal cancer resection	Colorectal cancer	Drug: saline Drug: ketamine	Department of Anesthesiology and Pain Medicine, Yonsei University College of Medicine, Seoul, Republic of Korea, 03772

**Table 2:** Clinical application of sevoflurane in the treatment of cancers.

Status	Study Title	Condition	Interventions	Locations
Recruiting	The effects of propofol-based intravenous vs. sevoflurane inhalation anaesthesia on inflammation and circulating tumor cells in paediatric tumor surgery—a pilot study	Solid tumor Carcinoma Malignancy Cancer	Drug: propofol Drug: sevoflurane	Hong Kong Children’s Hospital, Hong Kong, China.
Recruiting	The influence of anesthesia on postoperative outcome and complications in colorectal cancer patients	Colorectal cancer	Drug: TIVA + lidocaine Drug: sevoflurane + lidocaine Drug: TIVA + Placebo Drug: Sevoflarane + placebo	Clinical ATI, Str Crotoiilor nr 19-21, Clusnapoca, Cluj, Romania, 400162
Recruiting	Contribution to the elucidation of the mechanisms and effects by which certain perianesthetic interventions modify long-term evolution of patients with digestive cancers subjected to surgery	Colorectal cancer	Drug: Lidocaine 1% injectable solution Biological: blood extraction Drug: sevoflurane Drug: propofol	Institutu Regional de Gastroenterologie si Hepatologi Cluj-Napoca, Cluj, Romania, 400469

**Table 3:** Summary of the anti-cancer effects of the anesthetics.

Year	Study Design	Cell Lines/Animal	Anesthetics	Pathways	Major Findings	Ref.
2014	In vitro	MA-10 mouse Leydig tumor cell line	Midazolam	Caspase cascade, p-Akt pathway, P-38, and JNK pathways	Midazolam induces MA-10 cell apoptosis via activation of the caspase cascade, the inhibition of p-Akt, P-38, and JNK pathways.	[31]
2016	In vitro	MA-10 mouse Leydig tumor cell line	Midazolam	Apoptosis pathway, autophagy, and ER stress	Midazolam induces apoptosis of MA-10 cells through the induction of ER stress, regulation of cell cycle, and autophagy.	[32]
2018	In vitro	Human A549 non-small cell lung carcinoma	Midazolam	Mitochondria intrinsic apoptosis pathway, miR-520d-5p, STAT3 pathway	Midazolam induces apoptosis of A549 cells via induction of miR-520d-5p-inhibited STAT3 signaling.	[33]
2013	In vitro and xenograft model	Human K562 leukemia cells and HT29 colon cancer cells	Midazolam	Cell cycle, Intrinsic apoptosis pathway, ERK pathway, reactive oxygen species	Midazolam inhibits proliferation and induces mitochondria intrinsic apoptotic signaling. Midazolam also inhibits HT29 tumor growth in a mouse xenograft model. The mechanisms involve the inhibition of ROS, induction of apoptosis, and inhibition of growth-related proteins.	[34]
2020	In vitro	Human hepatocellular carcinoma cell, HepG2	Midazolam	Cell migration, invasion, proliferation, apoptosis	Midazolam inhibits cell proliferation and promotes apoptosis by inducing microRNA miR-124-3p	[35]
2010	In vitro	Human Jurkat T lymphoma cell, SHEP neuroblastoma cell, and primary rat cortex neurons	Midazolam	The mitochondrial intrinsic apoptotic pathway	Midazolam induces apoptosis of all cell types through the induction of caspase 9 and suppression of BCL-2, whereas deficiency of FADD and caspase 8 has no effect.	[36]
2021	In vitro	Human glioblastoma cell lines U118-MG andU87MG	Remimazolan	Apoptotic pathway, NF-κB pathway	Remimazolan induces apoptosis of glioblastoma cells through the activation of caspase cascade, inhibition of NF-κB, and downstream anti-apoptotic protein, such as XIAP and survivin.	[38]
2018	In vitro and xenograft model	Human A549 non-small cell lung carcinoma, H4 neuroglioma cell line	Midazolam, Dexmedetomidine	The mitochondrial intrinsic apoptotic pathway, PBR pathway, cell migration	Dexmedetomidine promotes cancer progression in both cell lines, whereas midazolam inhibits cancer progression by induction of mitochondrial intrinsic apoptotic pathway. The inhibitory effect of midazolam is partly mediated by PBR.	[39]
2021	In vitro	Human A549 non-small cell lung carcinoma, breast cancer cell line MCF-7, MDA-MB-231 human breast cancer cell line	Midazolam	Cell migration, invasion, epithelial-mesenchymal transition (EMT), PBR	At low dosage, midazolam treatment inhibits TGF-β-induced proliferation, migration, invasion, and EMT in A549 and MCF-7 through PBR. Midazolam inhibits proliferation, migration, invasion, and mesenchymal marker of MDA-MB-231 cells.	[40]
2020	In vivo	Hyperglycemia-induced pulmonary vascular leakage and cancer metastasis in diabetic mice	Midazolam	Lung metastasis, reactive oxygen species, endothelium leakage	Subcutaneous injection of midazolam inhibited hyperglycemia-induced cancer metastasis in the lungs of diabetic mice by preventing the generation of ROS, activation of transglutaminase, and subsequent vascular leakage.	[41]
2021	In vitro and xenograft model	Cisplatin-resistant non-small cell lung cancer cell line	Midazolam	Proliferation, apoptosis, microRNA	Midazolam suppresses cell proliferation and viability and promotes cell apoptosis in cisplatin-treated CR-NSCLC cells. Midazolam enhances cisplatin sensitivity in CR-NSCLC cells via modulating the miR-194-5p/hook microtubule-tethering protein 3 (HOOK3) axis.	[42]
2022	In vitro and transgenic mouse model	Murine pancreatic ductal adenocarcinoma cell lie, transgenic PDAC mouse model (*LSLkras^G12D/+^; Trp53^flox/flox^;Pdx-1^cre/+^* [*KPPC*])	Midazolam	Anti-tumor, inflammatory response	Midazolam significantly inhibited tumor size and proliferative index of Ki-67 and cyclins in PDAC through PBR. Midazolam inhibits cancer-associated neutrophils, macrophages, PMN, and pro-inflammatory cytokines through PBR.	[45]
2012	In vitro	human neuroblastoma cell line SN-K-SH	Haloperidol	Apoptotic pathway	Haloperidol, but not risperidone and paliperidone, induces neuroblastoma cell death via induction of apoptosis	[48]
2020	In vitro	Human glioblastoma (GBM) U87, U251, and T98 cell lines	Haloperidol	Cell cycle, cell migration, apoptotic pathway	Treatment with haloperidol reduces the viability of these GBM cell lines by induction of apoptosis. Haloperidol inhibits cell migration and CD24/CD44 alteration expression. Haloperidol, combined with TMZ and radiation therapies, further increased tumor cell death.	[51]
2009	In vitro	PC12 rat pre-neuronal cell line	Haloperidol	Akt pathway, mitochondria intrinsic apoptotic pathway	Haloperidol inactivates Akt, which induces the dephosphorylation of serine in Bcl-XS and promotes its association with the mitochondrial voltage-dependent anion channel (VDAC), and with cytochrome *c*- and caspase-3-dependent events.	[52]
2020	In vitro	human ovarian cancer cell lines OVCAR-3, SKOV3, A2780, 3AO, COC1, OV-90	Ketamine	Cell proliferation, apoptotic pathway, long noncoding RNA, histone acetylation, and methylation	Ketamine inhibits the proliferation and survival of six ovarian cancer cell lines by regulating P300-mediated H3K27 acetylation activation in the promoter of PVT1, which in turn binds EZH2 and promotes p57 expression.	[62]
2021	In vitro and xenograft model	Human breast cancer cell lines MCF-7 and T47D; Human liver cancer cell (HCC) lines HepG2 and Huh7	Ketamine	Apoptosis, ferroptosis, long noncoding RNA (lncRNA), reactive oxygen species	Ketamine suppresses the viability and proliferation of liver and breast cancer cells through the activation of ferroptosis. Ketamine-induced ferroptosis is mediated by the inhibition of lncPVT1 and glutathione peroxidase 4 (GP4) in HCC, whereas ketamine induces the levels of MDA, lipid ROS, and Fe^2+^ and attenuates the KAT5-mediated decrease of GP4 in breast cancer cells.	[64,65]
2016	In vitro	Human ovarian carcinoma (SKOV-3) and prostate carcinoma (PC-3)	Bupivacaine	glycogen synthase kinase-3β, apoptosis	Bupivacaine reduces cell viability and inhibits proliferation and migration but induces apoptosis of both cell lines. Bupivacaine increased the phosphorylation of GSK-3β^Tyr216^ in SKOV-3, which promotes the sensitivity of ovarian cancer cells to bupivacaine-induced cytotoxicity.	[71]
2021	In vitro	Human ovarian cancer cell line SKOV3 and COC1	Lidocaine	Proliferation, apoptosis, Wnt pathway	Lidocaine inhibits proliferation, migration, and invasion, and induces apoptosis in ovarian cancer cell lines. Overexpression of Wnt/β-catenin signaling overcomes lidocaine-inhibited cell migration and invasion.	[72]
2017	In vitro and xenograft model	Human liver cancer cell (HCC) lines HepG2	Lidocaine	Cell cycle, mitochondria intrinsic apoptotic pathway, MAPK pathways	Lidocaine inhibits the growth but stimulates apoptosis of HepG2 through ERK/P38-mediated mitochondria intrinsic apoptotic pathway.	[73]
2018	In vitro	Human prostate cancer cell line DU-145	Levobupivacaine	Glycolysis, oxidative phosphorylation, reactive oxygen species, cell cycle	Levobupivacaine induces inhibition of glycolysis and oxidative phosphorylation in cancer cells, which in turn decreases cellular ATP production and consecutive bioenergetic crisis, together with reactive oxygen species generation. Cancer cells are arrested in the S phase without triggering apoptosis.	[74]
2021	In vitro and xenograft model	Human non-small cell lung cancer cell lines, A549 and A427	Levobupivacaine	Ferroptosis, proliferation, apoptosis, reactive oxygen species, P53	Treatment of levobupivacaine increases the levels of ROS, iron, Fe^2+^, and ferroptosis but attenuates migration and invasion of NSCLS. The levobupivacaine-induced ferroptosis is mediated by the regulation of P53.	[75]
2021	In vitro and xenograft model	Human gastric cancer cell lines, HGC27 and SGC7901	Levobupivacaine	Ferroptosis, proliferation, microRNA	Treatment of levobupivacaine increases the levels of Fe^2+^/iron and lipid ROS and ferroptosis in erastin and RSL3-stimulated gastric cancer cells. This levobupivacaine-induced ferroptosis is mediated by upregulation of miR-489-3p and then targeting SLC7A11.	[76]
2003	In vitro	Human breast cancer cell line MCF-7	Procaine	DNA methylation, proliferation	Procaine can demethylate densely hypermethylated CpG islands, restoring gene expression of epigenetically silenced genes and inhibiting cancer proliferation.	[77]
2021	In vitro	Human gastric cancer cell line SGC-7901	Propofol	Proliferation, microRNA	Propofol inhibits the proliferation of gastric cancer cells by upregulating the has-miR-328-3p, which then downregulates the downstream genes, such as STAT3, MMP2, CCND1, and COX2.	[86]
2019	In vitro	MA-10 mouse Leydig tumor cell line	Propofol	Proliferation, mitochondria intrinsic apoptosis pathway, MAPK pathways, Akt pathways	Propofol decreases cell viability and increases mitochondria intrinsic apoptosis pathway. This apoptotic induction may be regulated by the MAPK activation and inhibition of Akt phosphorylation.	[88]
2021	In vitro	Human oral squamous cell carcinoma cell lines, SAS, SCC9	Propofol	Apoptosis, drug- resistance, growth factors	Propofol decreases cell viability and promotes cell apoptosis. The expression and activation of amphiregulin is related to 5-FU resistance, where propofol ameliorates 5-FU drug resistance by downregulation of amphiregulin.	[89]
2008	In vitro and xenograft model	Human cervical cancer cell lines HeLa, Ca Ski and SiHa	Valproic acid (VPA)	Proliferation, histone acetylation, angiogenesis	VPA induces histone H3 acetylation and upregulates p21, cytostatic effects both in vitro and in vivo. VPA can also inhibit angiogenesis in vivo.	[94]
2006, 2012	In vitro and xenograft model	Human prostate cancer cell lines LNCaP, PC3, and DU145	Valproic acid (VPA)	Proliferation, histone acetylation, cell cycle, apoptosis	Chronic administration of VPA decreases prostate cancer cell net proliferation with increased caspase activation both in vitro and in vivo.	[95,96]
2020	In vitro, xenograft model, and human samples	Human gastric cancer cell line SGC-7901	Valproic acid (VPA)	Autophagy, apoptosis, histone deacetylase (HDAC), Akt pathways	Treatment of VPA inhibits HDAC1/2 activity and induced autophagy and then apoptosis. HDAC1/PTEN/Akt pathway and the regulation of BCL-2 and beclin-1 are involved in the inhibitory effects of VPA. The expression of HDAC correlates with poor prognosis in human gastric patients.	[97]
2015	In vitro	Human breast cancer cell lines, MCF-7 and SKBR3, MDA-MB-231, and BT474	Valproic acid (VPA)	Proliferation, histone acetylation, heat shock protein, protein acetylation	Treatment of VPA inhibits proliferation of four breast cancer cell lines and with better inhibition in HER2-overexpressing SKBR3. VPA can also upregulate expression of p21 WAF1, cleaved caspase-3, and acetylation of heat shock protein 70.	[99]
2020	In vitro	Human sorafenib-resistant hepatocellular carcinoma cell line, HepG2-SR	Valproic acid (VPA), sorafenib	Drug resistance, Notch pathway, Akt pathway	Notch1 and Akt are upregulated in sorafenib-resistant cells. The combination of VPA and sorafenib treatment enhances sensitivity of drug-resistant cells and reverse the increased levels of Notch1 and Akt in HepG2-SR.	[101]
2019	In vitro	human prostate tumor cell lines PC3, DU-145, and LNCaP	Valproic acid (VPA)	Drug resistance, Akt-mTOR pathway, cell cycle, histone acetylation	In Temsirolimus resistant cell lines, the elevation of cell proliferation and clonal growth is associated with cell cycling proteins cdk1 and cyclin B, along with increases in Akt-mTOR signaling but decreases in p19, p21, and p27. Treatment of VPA inhibits cell growth and upregulates the acetylated histones H3 and H4 together with the decrease of Cdk1 and cyclin B phosphorylation of mTOR and the mTOR sub-complex Raptor.	[104]
2019	In vitro	Human glioblastoma cell lines, U251 and U87	Sevoflurane	Proliferation, invasion and migration, apoptosis, insulin-like growth factor (IGF-1) pathway, PI3K/Akt pathway.	Sevoflurane treatment inhibits proliferation, migration, and invasion but promotes apoptosis in glioblastoma cell lines. This inhibitory effect of sevoflurane is mediated by IGF-1/PI3K/Akt signaling.	[116]
2019	In vitro and xenograft model	Human colon cancer cell line SW480 and SW620	Sevoflurane	Proliferation, cell cycle, apoptosis, autophagy, invasion, and epithelial-mesenchymal transition	Sevoflurane treatment inhibits proliferation, invasion, and cell cycle progression, and promotes apoptosis and autophagy through Raf/MEK/ERK pathways.	[117]
2019	In vitro, human samples	Human colorectal cancer (CRC) cell lines SW620 and HCT116	Sevoflurane	Migration and invasion, ERK/MMP pathway, microRNA	Sevoflurane treatment inhibits migration and invasion but not the proliferation of CRC cell lines. Treatment of sevoflurane downregulates phosphorylation of ERK (p-ERK) but restores expression of miR-203. Inhibition of miR-203 attenuates the inhibitory effect of sevoflurane on cell migration, invasion, and p-ERK.	[118]
2018	In vitro	Human breast cancer cell lines, MCF-7 and MDA-MB-231	Sevoflurane	Proliferation, cell cycle, microRNA	Treatment of sevoflurane inhibits the proliferation of breast cancer cell lines by activating miR-203.	[119]
2015	In vitro	Human non-small cell lung carcinoma cell line, A549	Sevoflurane	Hypoxia, proliferation, metastasis, P38 MAPK	Sevoflurane treatment suppresses hypoxia-induced proliferation and metastasis of A549 cells by modulating HIF-1α and its downstream genes. In addition, the p38 MAPK pathway is involved in regulating HIF-1α by sevoflurane.	[120]
2018	In vitro, xenograft model, and human samples	Primary culture of human hepatocellular carcinoma	Isoflurane	Proliferation, apoptosis, migration and invasion, PI3K/Akt pathway, NF-κB pathway	Treatment of isoflurane inhibits growth and decreased viability of liver cancer cells in vitro and in vivo, and the apoptotic rate is increased in cells obtained from isoflurane-treated patients. Treatment of isoflurane inhibits PI3K/Akt to regulate cell survival, whereas isoflurane-attenuated NF-κB inhibits migration and invasion of cancer cells.	[121]

## Data Availability

Not applicable.
